# Controlled versus Automatic Processes: Which Is Dominant to Safety? The Moderating Effect of Inhibitory Control

**DOI:** 10.1371/journal.pone.0087881

**Published:** 2014-02-10

**Authors:** Yaoshan Xu, Yongjuan Li, Weidong Ding, Fan Lu

**Affiliations:** 1 Key Laboratory of Behavioral Science, Institute of Psychology, Chinese Academy of Sciences, Beijing, PR China; 2 University of Chinese Academy of Sciences, Beijing, PR China; 3 Research Institute of Nuclear Power Operation of China, Wuhan, Hubei, PR China; University of Missouri-Kansas City, United States of America

## Abstract

This study explores the precursors of employees' safety behaviors based on a dual-process model, which suggests that human behaviors are determined by both controlled and automatic cognitive processes. Employees' responses to a self-reported survey on safety attitudes capture their controlled cognitive process, while the automatic association concerning safety measured by an Implicit Association Test (IAT) reflects employees' automatic cognitive processes about safety. In addition, this study investigates the moderating effects of inhibition on the relationship between self-reported safety attitude and safety behavior, and that between automatic associations towards safety and safety behavior. The results suggest significant main effects of self-reported safety attitude and automatic association on safety behaviors. Further, the interaction between self-reported safety attitude and inhibition and that between automatic association and inhibition each predict unique variances in safety behavior. Specifically, the safety behaviors of employees with lower level of inhibitory control are influenced more by automatic association, whereas those of employees with higher level of inhibitory control are guided more by self-reported safety attitudes. These results suggest that safety behavior is the joint outcome of both controlled and automatic cognitive processes, and the relative importance of these cognitive processes depends on employees' individual differences in inhibitory control. The implications of these findings for theoretical and practical issues are discussed at the end.

## Introduction

Despite attempts to improve workplace safety, high rates of job-related accidents and injuries persist worldwide [1], [2]. Approximately 120 million workplace-related accidents occur every year, resulting in 200,000 fatalities and 4%–5% loss of gross domestic product worldwide [3]. In China alone, 79,552 workers lost their lives in 363,383 industrial accidents in 2010 [4].

Unsafe behaviors of employees (i.e., violations) are often cited as the triggers of accidents and injuries [5]. In addition, since pre-existing hazardous conditions can also contribute to accidents [6], it is important to promote behaviors that can reduce such hazards and maintain safe working conditions. Researchers have distinguished two types of safety behaviors — namely, safety compliance and safety participation [7] — that directly predict accidents and injuries [2]. Safety compliance refers to the behavior focused on meeting minimum safety standards at work, such as compliance with safety procedures, which is the opposite of violation (noncompliance) [8]. Safety participation refers to voluntary safety behaviors, such as helping coworkers with safety-related issues [9].

Significant interest has been shown in identifying social cognitive factors that can predict employees' safety behaviors [10]–[12]. The dual-process theory suggests that there are two distinguished cognitive processes, namely, controlled processes and automatic processes (also called controlled cognition and automatic cognition, respectively) that play roles in predicting behavior [13]–[18]. Previous safety studies have drawn attention primarily to the former, whereas the latter has been relatively neglected in spite of its unique contribution to behavior [19]–[21]. Recently, research has begun to examine the role of automatic cognition in safety studies [22], [23]. The first aim of the present study is to examine whether controlled cognition together with automatic cognition can better predict safety behaviors. In addition, considering that executive function has been suggested to be a boundary condition for both of these cognitive processes in driving social behaviors [24], [25], our second aim is to examine whether individual differences in inhibition (a core component of executive function) can shift the relative weight of controlled and automatic cognitive processes in predicting employees' safety behaviors in the workplace.

### What is the Dual-process Theory?

Over the past two decades, dual-process models have been used in important accounts of human behavior across various fields of psychology. Dual-process models assume that human behaviors are generally the result of two different modes of information processing: controlled and automatic processes [13]–[18]. Controlled cognition is slow, intentional, explicit, and effortful awareness that influences human behavior in a deliberate analytical way [14]–[16]. In addition, people have the appropriate intention and carry out deliberate behaviors by retrieving information from memory with effort in the controlled process [16], [26]. Generally, controlled cognition is measured via traditional verbal self-reports [26].

In contrast, automatic cognition is fast, unintentional, implicit, and out of awareness [17], [18]. Most commonly, automatic cognitions are depicted as unconscious mental associations between concepts and valences in an associative network or habitual responses that can generate quick spontaneous behavioral tendencies without intention [21, 27 28, 29]. For example, the automatically activated associations between “candy” and “pleasure” may induce an impulse to consume candy without deliberate thought and intention. Automatic cognitions are usually measured by implicit or indirect measures, such as implicit association tests (IATs) [30], [18]. Since such measures typically use reaction latencies to tap into aspects of automatic information processing, they do not necessarily hinge on participants' abilities and willingness to report a given construct [31].

### Safety Attitude and Controlled Cognitive Process in Workplace Safety

In the workplace safety domain, many studies have tried to explain safety behaviors from the perspective of employees' attitudes concerning organizational safety issues [32]–[35]. Based on the general definition of attitude, attitude toward safety is a psychological evaluation of safety issues captured in attribute dimensions like important-unimportant, necessary-unnecessary, and harmful-beneficial and so on [26]. Based on the general assumption that attitude toward an object can lead to behaviors that sustain the object [36], an individual's evaluation of safety issues is expected to have a direct effect on the individual's safety behaviors. Empirical studies have shown that employees' self-reported evaluations regarding safety issues are positively associated with safety behaviors [24], [37]. And individuals with higher tolerance for unsafe behaviors display more risky and noncompliant behaviors [33], [38].

Typically, employees are asked to verbally report their explicit and conscious evaluations about safety issues, which reflect employees' controlled cognitive process regarding safety. According to this process, behavior is deliberate and intentional; that is, employees need to make an analytical, effortful evaluation of safety issues before engaging in safety behaviors [34], [35].

### Why is Automatic Cognitive Process Important in Workplace Safety?

In recent years, researchers have begun to draw attention to the role of automatic cognitive processes in the context of safety [22], [23], [39], [40]. There are several reasons for considering automatic cognitive processes in the safety domain. First, drawing on Schein's (1990) model of organizational culture [41], which proposed “three interconnected levels of culture: observable artifacts, espoused values, and basic underlying assumptions”, Burns et al. (2006) distinguished surface levels and deeper levels of safety culture [22]. This model suggested that cognitions that belong to the surface level are conscious and explicit attitude, while those that belong to the deeper level reveal the “basic underlying assumption” about safety and are unconscious and automatic [41]. Therefore, investigating both controlled and automatic cognitive processes is important for a full understanding of cognition related to safety.

Second, since employees cannot exert full effort to control their behaviors at every moment [36], safety behaviors may consists of elements of deliberative and sustained efforts, as well as behaviors that are discretionary and automatic [22]. Consistent with this hypothesis, a study found that work habits and behavioral intentions jointly contribute to food safety behavior [39]. An automatic association between the concept of “I” and the attribute of “carefulness” was also found to be a complementary predictor of safety performance [23]. Further, an automatic association between flying behaviors (safe flight vs. risky flight) and attributes (pleasure vs. displeasure) predicted flying performance in a simulated flying task [40].

In addition, from a methodology perspective, automatic cognition is less influenced by deliberate cognitive and motivational forces such as social desirability compared to self-reported measurements of controlled cognition [42]. Since safety is a sensitive issue in a safety-priority organization, when asked directly, employees may be more likely to report the importance of safety [22], [23]. In indirect measures, participants have no control of the measurement outcome [43]; thus, response biases can be avoided to some extent [30].

### Automatic Associations towards Safety and Safety Behaviors

In the safety field, the Expectancy-valence Theory predicts that employees will be motivated to adhere to safety policies and participate in safety activities if they have positive safety behavior-outcome expectancies [44]–[46]. Specifically, if employees are rewarded for improving safety and punished for taking risks, their motivational force to safety behaviors would be high. On the contrary, if employees are not punished or even rewarded for risky behaviors (e.g., saving time), their motivation to safety behaviors would be low. With repeated pairings of safe or risky activities with positive or negative outcomes in everyday work, associations of safety and risk with positive and negative valences, respectively, would be formed in the mental associative network. This process is automatic, spontaneous, and can generate corresponding behavioral impulses without deliberate effort [21]. For example, a strong automatic association between safety and positive valence and risk and negative valence can induce a behavioral tendency to sustain or improve safety and avoid or reduce risk.

Actual behaviors can be directly induced by the impulses generated by automatic association without effort and intention at some moments in daily work [22], [36]. Employees who strongly associate safety with positive valence and risk with negative valence may comply with safety procedures and participate in safety activities spontaneously under the impulse to maintain/improve safety and to avoid/reduce risk. On the contrary, employees who weakly associate safety with positive valence and risk with negative valence may be more likely to engage in violation and be less motivated to participate in extra safety activities. Therefore, we expect that automatic association concerning safety should drive both safety compliance and safety participation:

*Hypothesis 1: Automatic associations concerning safety will positively predict safety compliance and safety participation.*

### How does Inhibitory Control Shift the Relative Weight of Controlled and Automatic Processes?

Inhibitory control processes have recently been found to moderate the relative weights of controlled and automatic cognition on behavior in social psychology research [24], [47]. Inhibition is the ability to inhibit an inappropriate response or behavior impulse [25]. An individual with low levels of inhibition may tend to express more automatic racial prejudice [48], [49] and is less likely to resist spontaneous behavioral tendencies in eating [24] and drinking [50], [51]. By contrast, participants with high inhibitory control are more capable of executing deliberate behavioral intentions [52].

The moderating effect of inhibition can be interpreted in terms of the dual processes of behavior regulation [47]. Controlled cognitive process can sometimes adjust, interrupt, or even override and prevent actions driven by automatic cognitive process [29], [53]. Hence, an individual with high inhibitory control can inhibit the behavior an impulse generated by the automatic activation of association, and instead performs a more deliberate behavior, which is driven by delayed controlled cognition. By contrast, an individual with low inhibitory control is incapable of resisting spontaneous behavioral tendencies, so their behavior is influenced more by automatic cognitive processes. Based on these assumptions, we hypothesize that the influences of controlled and automatic cognitions on safety behavior depend on the individuals' level of inhibitory control:

*Hypothesis 2: The influence of controlled cognition on safety compliance and safety participation is stronger in employees with high inhibitory control than in employees with low inhibitory control. By contrast, the influence of automatic association on safety compliance and safety participation is stronger in employees with low inhibitory control than in employees with high inhibitory control.*

## Materials and Methods

### Ethics Statement

The Ethics Committee of the Institute of Psychology of the Chinese Academy of Sciences approved this research and the consent procedure. Given that the present study only measured simple behavior responses, it did not elicit adverse physiological and psychological reactions from the participants. Thus, the institutional ethics committee waived the need for written informed consent from the participants. After all procedures had been explained by one of the researchers, those participants who decided to join the study could get an invitation code to access the program, which was the representation of their oral consent. Participants had the right to withdraw from the study at any time, and the researcher counted four participants who quitted from the study in total. Approval of the study was also done by administrators of the nuclear power plant at which this study was conducted.

### Participants

One hundred and eight male control room operators of one nuclear power plant in China participated in this research. Four participants withdrew from the study. Five participants were excluded because of unreliable IAT scores (see below). Thus, data from 99 participants are included in this report. The age of the participants ranged from 25 to 30 years (*M* = 28.28, *SD* = 2.01), and their tenures ranged from 2 to 6 years (*M* = 4.28, *SD* = 1.00). Age and tenure were not significantly correlated with safety compliance (*r* = −0.05, *r* = −0.09) and safety participation (*r* = 0.01, *r* = 0.03).

### Procedure

Before the study, the operators were informed that a study was being conducted to identify the factors affecting operators' performances and that they were welcome to join the research as volunteers. They could select any day of the designated week to go to the computer room at their training center to provide sample data in their spare time. When participants sat in front of the computer, the whole procedure of the study was explained to them. First, the purpose of the research was introduced in detail. Secondly, the duration of the study, the measures involved (implicit association tests and questionnaires); the involved researchers and their affiliations were introduced. Finally, participants were promised that they would remain anonymous and their data would be held in confidentiality. They were also informed that they were free to withdraw from the study at any time, or to decline to answer any particular question in the study. After agreeing to join the study, each participant was assigned a unique invitation code to access the program.

Then, the IAT was performed, followed by the Stroop task. Instructions for the two tests were given on the computer screen before the tasks were initiated. Subsequently, the participants filled out paper-and-pencil questionnaires in another room. Safety behaviors were assessed after safety attitudes and demographic data were collected last. Finally, the participants were thanked for taking part in the study.

### Study Measures

Participants were asked to rate their safety behaviors during the past half year. Two components of safety behavior were assessed: safety compliance and safety participation. Each safety component was assessed by three items [7]. A sample item for safety compliance is “I use all the necessary safety equipment to do my job” (α = 0.81); a sample item for safety participation is “I promote the safety program within the organization” (α = 0.85). Responses were recorded on a five-point scale ranging from 1 (never) to 5 (frequently). A confirmatory factor analysis (CFA) of the two dimensions of safety behavior resulted in acceptable fit (χ2/df = 17.50/8 = 2.19, *p* = 0.03, GFI = 0.95, NFI = 0.94, CFI = 0.96, RMSEA = 0.11).

The controlled cognitive process regarding safety was measured using responses of the safety attitude questionnaire [54]. General safety attitude and safety compromise, two dimensions that directly measure employees' concerns about safety, were used in the current study. All items were measured on a 6-point rating scale ranging from 1 (strongly disagree) to 6 (strongly agree). General safety attitude was assessed based on responses to questions regarding safety issues in the workplace (α = 0.78) (see Table S1). A sample item is “Safety specific jobs should always get done.” To measure safety compromise, three questions that assessed attitudes about risky activities at work in favor of productivity were used (α = 0.65) (see Table S1). A sample item is “Sometimes it is necessary to take risks to get a job done.” CFA of the two dimensions of safety attitude resulted in a satisfactory fit (χ^2^/*df* = 20.33/14 = 1.45, *p* = 0.12, GFI = 0.95, NFI = 0.92, CFI = 0.97, RMSEA = 0.07).

Automatic association was measured by a computerized IAT task, which provides an indirect measure of the strength of automatic associations of two categories (i.e., safety vs. risk) with two attributes (positive vs. negative). The underlying logic of IAT is that the strength of automatic associations between objects and attributes can be reflected by differences in participants' reaction time in sorting target objects with positive and negative attributes. When an object and an attribute that share the same response are closely associated to an individual, the sorting task is easier than when an object and an attribute sharing a response are weakly associated. In the current study, if someone strongly associates safety with positive valence and risk with negative valence, they will find the classification task easier when safety is assigned the same key response as positive words and risk is assigned the same key response as negative words than the opposite condition. Therefore, the difference in response times between the two conditions is taken as an indicator of the degree of association between mental concepts [30].

In the IAT task, one stimulus (“safety”) was used in the “safety” category, and one stimulus (“risk”) was used in the “risk” category. Although this task generally involves use of two or more words per category, a 1-word-per-category procedure is justifiable in the current study since it is difficult to find other words that unambiguously represent the category “risk”. A similar one-word-per-category procedure has been used successfully in a previous study to measure implicit risk association [55]. The attribute stimulus consisted of seven positive words (labeled “positive”) and seven negative words (labeled “negative”), which were consistent with those of a previous study [55] (see Table S2). The participants were told that a target word would be displayed in the center of the screen and their task would be to classify each word by using the left (“f” key) or right (“j” key) key as fast as they could while avoiding errors. The computer recorded the elapsed time between the start of each stimulus presentation and the correct response. If the participant made an incorrect categorization, an “Error” would be displayed on the screen as a warning until the correct response was made.

The task was comprised of five phases. The first phase was initial target concept discrimination (e.g., participants press the left response key when the target is “safety” and the right response key when the target is “risk”). The second phase was attribute discrimination (e.g., participants press the left response key when the targets are positive expectancy words and the right response key when the targets are negative expectancy words). The third phase was the first combined task (e.g., participants press the left response key when the targets are “safety” or positive expectancy words and the right response key when the targets are “risk” or negative expectancy words). The fourth phase was the reversed target–concept discrimination (e.g., participants press the left response key when the target is “risk” and the right response key when the target is “safety”). The fifth phase was the reversed combined task (e.g., participants press the left response key when the targets are “risk” or positive expectancy words and the right response key when the targets are “safety” or negative expectancy words). Each single-dimension discrimination block (i.e., Phases 1, 2, and 4) consisted of 30 trials, and each combined discrimination block (i.e., Phases 3 and 5) consisted of 60 trials. Words were displayed randomly for each participant. The inter-trial interval was 400 milliseconds. The order of the two combined conditions and left-right placement were balanced between the subjects.

Consistent with data reduction procedures described in a previous study [36], data from participants who had average response latencies under 400 milliseconds combined with error rates over 40% were omitted (n = 5) before data analyses were conducted. These procedures were employed to eliminate participants who did not take the task seriously or did not understand the nature of the task. For the remaining participants, two mean reaction times were calculated: the first mean reaction time covered the condition in which safety shared a key with positive words and risk shared a key with negative words (compatible condition), and the second mean reaction time covered the condition in which safety shared a key with negative words and risk shared a key with positive words (incompatible condition). The index of IAT (IAT D score) was computed by dividing the mean latency difference between incompatible and compatible conditions by their overall standard deviation (SD) [56]. If someone associates safety with positive words and risk with negative words more strongly than safety with negative words and risk with positive words, it is easier for them to execute the classification task under the compatible condition than that under the incompatible condition. Therefore, a large IAT D score would denote that an individual strongly associates safety with positive attributes and risk with negative attributes.

Inhibitory control was measured by using the computerized Stroop task [57]. In this task, participants were instructed to name the color of a word as quickly as possible in each trial by pressing the stated keys. This procedure required the participants to inhibit or override the tendency to produce a more dominant or automatic response (e.g., name the word). The task was comprised of three conditions: the incongruent condition consisted of 48 trials with the name of a color printed in a different color (e.g., the word “BLUE” printed in red). The neutral condition contained 48 trials with a neutral word printed in four colors (e.g., the word “boat” printed in red). The congruent condition consisted of 48 trials with the name of a color displayed in its own color (e.g., the word “BLUE” printed in blue). Participants received a combination of the different trial types. Each trial consisted of a stimuli word presented in a colored script on a black background, which remained visible until the participant made a response or when 2 s had elapsed. An inter-trial interval of four seconds was used. The color-labeled response keys were “V,” “B,” “N,” and “M” for the colors red, blue, green, and yellow, respectively.

The response latencies of all participants were collected and trials with incorrect responses and with response latencies ±3 SD away from each participant's mean response latency were eliminated as outliers before further analyses. Two mean reaction times (corresponding to responses under incongruent and congruent conditions) were calculated. The index of inhibition was calculated as the mean difference in reaction time between the incongruent and congruent conditions (Stroop effect), with higher scores indicating lower inhibition.

### Statistical Analysis

Multiple moderated regression analysis was employed to test the main hypotheses. All variables were z-standardized prior to the regression analyses and computation of the interaction terms. Two four-step multiple regression analyses predicting safety compliance and safety participation were conducted. In step 1, the two dimensions of self-reported attitude concerning safety were entered. In step 2, the IAT was entered to test the main effect of automatic association regarding safety on safety behaviors (H1). In step 3, scores of the Stroop effect task were entered. In step 4, data on interaction between self-reported safety attitude and Stroop effect and that between IAT and Stroop effect were entered to examine the moderating effect of inhibition on the relationship between controlled/automatic cognitions and safety behaviors (H2).

## Results

### Initial analyses of IAT

Two mean reaction times from compatible (M = 781.44 ms, SD = 122.08) and incompatible conditions (M = 1051.60 ms, SD = 212.70) and the IAT effect (D measure) were calculated. The IAT effect was significantly different from zero (M = 0.72, SD = 0.54, *t* (98)  = 13.42, *p*<0.001), indicating that employees generally automatically associate safety with positive valance and risk with negative valence. However, significant individual differences do exist because the IAT effect ranges from −0.57 to 2.07, and the SD is relatively large (SD = 0.54).

### Preliminary Analyses

Table 1 presents the means, SD, and zero-order correlations of the study variables. The IAT score was not related to either dimension of self-reported safety attitude. Across all participants, general safety attitude was significantly positively related to safety participation, and safety compromise was significantly negatively related to safety compliance and safety participation. IAT was significantly positively related to safety compliance and safety participation. Scores on the Stroop task were not significantly related to any variable.

**Table 1:** Means, standard deviation, and Zero-order correlations of all variables (n = 99).

Variables	Mean	SD	1	2	3	4	5
1. General safety attitude	5.77	0.32	–				
2. Safety compromise	1.96	0.75	−.50^**^	–			
3. IAT	0.71	0.53	.12	−.11	–		
4. Stroop	141.72	86.63	−.01	−.01	−.12	–	
5. Safety compliance	4.74	0.38	.16	−.20^*^	.24^**^	−.01	–
6. Safety participation	4.20	0.73	.29^**^	−.27^**^	.21^*^	.06	.52^**^

*Note:* ^*^p<.05, ^**^p<.01.

### Hierarchical multiple regression analysis

**Figure 1:**
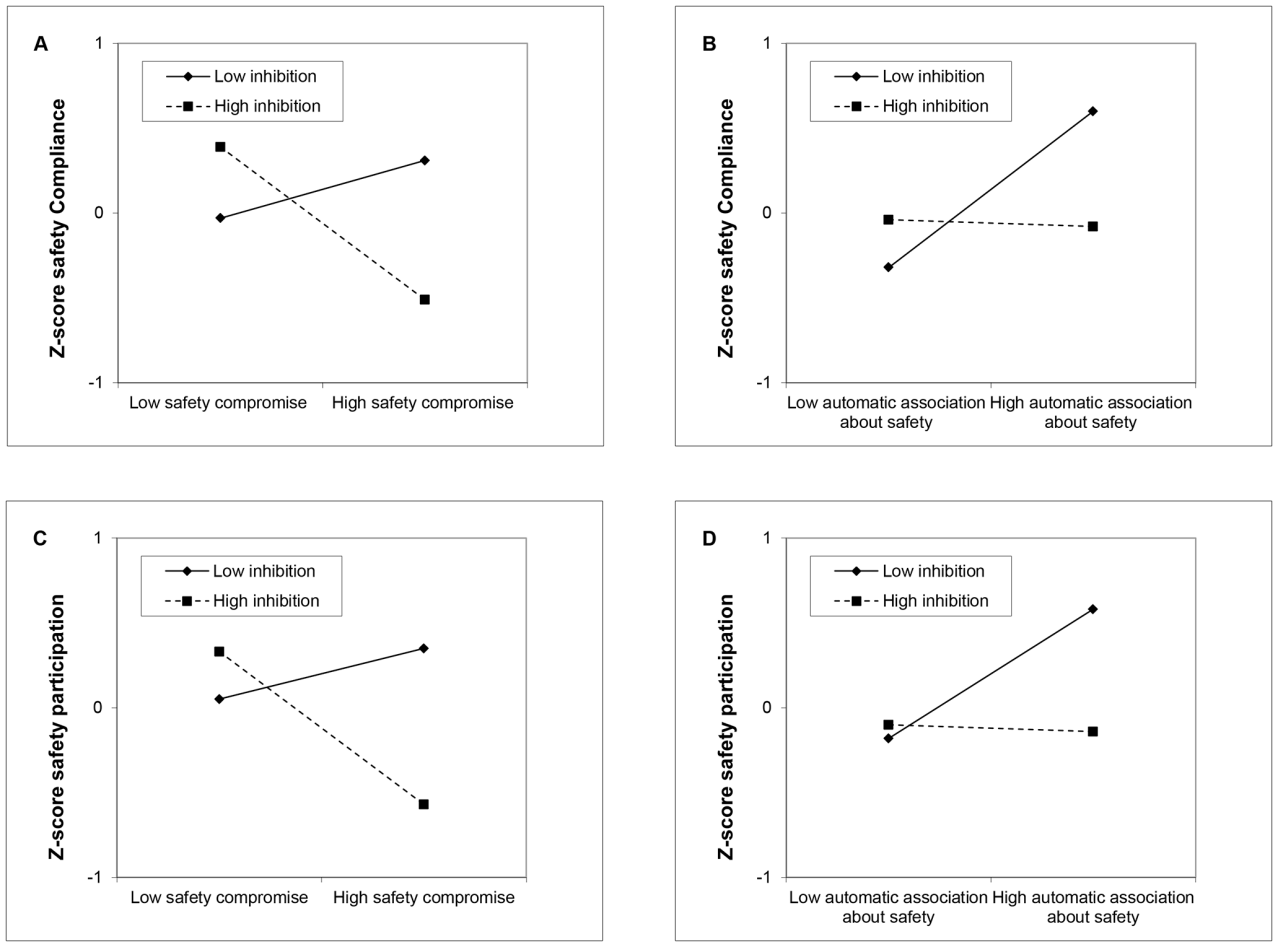
Moderating effects of inhibition on the influences of controlled and automatic cognitions on safety behaviors. Panel A shows the moderating effect of inhibition on the influence of safety compromise on safety compliance. Panel B shows the moderating effect of inhibition on the influence of automatic association on safety compliance. Panel C shows the moderating effect of inhibition on the influence of self-reported safety attitude on safety participation. Panel D shows the moderating effect of inhibition on the influence of automatic association on safety participation.

Results of a four-step multiple regression analysis predicting safety compliance (Table 2) showed that the two dimensions of self-reported attitude concerning safety did not have significant main effect on safety compliance. Instead, IAT was found to be significantly correlated with safety compliance (β = 0.22, *p*<0.05), which explained the observed 5% increase in variance. In addition, the scores of the Stroop effect did not predict safety compliance. The interaction between safety compromise and Stroop effect (β = 0.24, *p*<0.05), and that between IAT and Stroop effect (β = 0.31, *p*<0.01) significantly predicted safety compliance, explaining the observed 15% increase in variance. Specifically, safety compliance was negatively predicted by safety compromise among participants with high inhibitory control, while there was no significant effect of safety compromise on safety compliance in low inhibition participants (Figure 1a). Likewise, safety compliance was positively predicted by IAT among low inhibition participants. However, among those with high inhibitory control, there was no significant relation between IAT score and safety compliance (Figure 1b).

**Table 2:** Hierarchical multiple regression predicting self-reported safety compliance (n = 99).

Variables	Step 1	Step 2	Step 3	Step 4
General safety attitude	0.08	0.06	0.07	0.12
Safety compromise	−0.16	−0.15	−0.15	−0.14
IAT		0.22^*^	0.22^*^	0.22^*^
Stroop			0.02	0.10
General safety attitude × Stroop				−0.01
Safety compromise × Stroop				0.31^**^
IAT × Stroop				0.24^*^
F	2.33	3.28^*^	2.55^*^	4.23^**^
R^2^	0.05	0.09	0.09	0.25
ΔR^2^	0.05	0.05^*^	0.00	0.15^**^

*Note*: ^*^p<.05, ^**^p<.01.

Results of a multiple regression analysis predicting safety participation showed that general safety attitude was significantly correlated with safety participation (β = 0.23, *p*<0.05), while safety compromise did not have significant effect on safety participation (Table 3). After controlling for self-reported attitude, automatic association toward safety was positively and marginally significantly correlated with safety participation (β = 0.18, *p* = 0.06), revealing a trend in the predicted direction. The scores of the Stroop effect did not predict safety participation. The interaction between safety compromise and stoop effect (β = 0.20, *p*<0.05), and that between IAT and Stroop effect (β = 0.30, *p*<0.01) significantly predicted safety participation, and explained the 11% increase in variance. Specifically, safety participation was negatively predicted by safety compromise strongly among participants with high inhibition. However, among participants with low inhibition, there was no significant relationship between safety compromise and safety compliance (Figure 1c). For automatic association, the IAT score strongly predicted safety participation of participants with low inhibitory control, but there was no significant effect of IAT score on safety participation in high inhibition participants (Figure 1d).

**Table 3:** Hierarchical multiple regression predicting self-reported safety participation (n = 99).

Variables	Step 1	Step 2	Step 3	Step 4
General safety attitude	0.21	0.19	0.19	0.23^*^
Safety compromise	−0.16	−0.15	−0.15	−0.15
Automatic		0.17	0.18	0.18
Stroop			0.09	0.16
General safety attitude × Stroop				0.05
Safety compromise × Stroop				0.30^**^
IAT × Stroop				0.20^*^
F	5.44^**^	4.68^**^	3.70^**^	4.13^**^
R^2^	0.10	0.13	0.14	0.24
ΔR^2^	0.10^**^	0.03	0.01	0.11^**^

*Note*: ^*^p<.05, ^**^p<.01.

## Discussion

This study explored the precursors of employees' safety behaviors based on a dual-process model, which suggests that human behaviors are determined by both controlled and automatic cognitive processes. The relative importance of these two processes on safety behaviors was explored by examining the moderating effects of inhibition on the relationship between these two cognitions and safety behaviors. The results suggest that controlled cognitive processes (self-reported attitude) along with automatic cognitive processes (automatic associations toward safety) predicts employees' safety behaviors. In addition, the effect of controlled and automatic cognitive processes in determining safety behavior is moderated by employees' inhibitory control. The following sections focus on theoretical and practical implications, limitations of the present study, and direction of further research.

### Theoretical Implication

The current study tested the effect of controlled and automatic cognitive processes on safety behaviors as well as the moderating effects of inhibition. The results suggest that self-reported attitudes toward safety issues and the automatic associations about safety jointly predict employees' safety compliance and participation. This indicates that safety behaviors in the workplace are influenced by controlled as well as automatic processes, supporting Hypothesis 1. This result is consistent with previous literature in the organizational and safety domain [22], [23], [36], which show that job performance and safety performance comprises elements of deliberative and sustained efforts as well as discretionary, automatic, and impulsive behaviors. Therefore, the combination of controlled and automatic cognitive processes is helpful in better explaining safety behaviors in the workplace.

In addition, this study shows that the interactions between self-reported safety attitude and inhibition, and the interactions between automatic association and inhibition, explain safety behaviors, supporting Hypothesis 2. Interestingly, the signs of these interactions are in opposite directions. These results indicate that the relative weight of controlled and automatic cognitive processes in predicting safety behaviors depend on individual differences in response inhibition. Employees with low inhibition are more influenced by automatic cognitive processes, whereas controlled cognitive processes guide those with high inhibition. These interaction patterns are consistent with those in prior studies in several social psychological domains, including eating behavior, alcohol use, and stereotyping behavior [24], [31], [50], suggesting a dissociation between controlled and automatic cognitive processes [22], [47]. Hence, controlled and automatic cognitive processes influence safety behavior through different pathways. Automatic cognitive processes affect behavior through an impulsive and spontaneous process, through which employees' safety behavior are driven directly by the automatic impulse to improve safety and reduce risk generated by automatic association without intention and effort. However, controlled cognitive processes drive behavior through a deliberative and reflective process, according to which an automatic impulse is inhibited, and employees' safety behavior is guided by conscious thought and analysis. These results are also in accordance with the MODE (Motivation and Opportunity as Determinants of whether the attitude-to-behavior process is primarily spontaneous or deliberative in nature) which suggests that controlled cognitive processes predict more deliberative behaviors, whereas automatic cognitive processes predict more spontaneous behaviors [58].

### Practical Implication

From an applied perspective, the current study has several managerial implications. Most importantly, this study suggests the necessity of introducing automatic cognitive processes into safety management. First, safety managers can use indirect tests that capture employees' unconscious and automatic cognitions about safety [41], in combination with traditional self-reported measures to have a more comprehensive understanding of employees' critical cognitions related to safety in personnel selection.

Second, the results suggest that managers can encourage safety behavior by changing employees' automatic associations between safe/risk and positive/negative valences. Tracing the development of one's automatic association is difficult [23], [36] as any point of the work experience can influence its formation. Management should pay more attention to the circumstances (including words and deeds) surrounding the employees, especially newcomers. Changing of negative automatic association can be achieved by creating new automatic associations through an associative learning program [59], in which safety words can be presented as a repeat pairing with positive valence, and risk words can be presented as a repeat pairing with negative valence. Thus, strengthened associations occur between safety and positive behavior outcomes, which can in turn enhance safety performance through automatic activation of new associations.

Finally, given the significant effect of the interaction between attitude and inhibition on safety behavior, different safety training methods may be more effective for different subgroups of employees. On the one hand, employees with higher inhibitory control benefit from traditional interventions focused on changing controlled cognition, such as information-based techniques and feedback interventions [60]. On the other hand, employees with lower inhibitory control may benefit from interventions that attempt to strengthen the association between positive outcomes and desirable behaviors. Moreover, interventions should be aimed at improving self-regulation of impulsiveness for employees with strong association between risk and positive attributes but a lower level of inhibitory control. One such effective strategy is a self-control training regimen [61] that involves a physical regulation task. This strategy is effective in improving performance in self-control tasks. In safety-priority organizations such as a nuclear power plant, the training of employees' behavior during high workload and pressure (e.g., incidents with a simulator) is also important [21], [62]. Under these conditions, employees have limited opportunities and resources left to control their thoughts and behaviors, so the effect of automatic impulse on safety behavior may be stronger [47]. Such trainings can also help to adapt employees to emergencies and improve their ability to control their thoughts and behaviors under these situations.

### Limitations and Future Research

Considering some limitations of our results, they should be interpreted with caution. One of the limitations is the cross-sectional design, which places some restrictions on the prospective nature of these data. Further research employing a longitudinal design can help to determine whether the main effects of self-reported safety attitudes and automatic association, as well as the interactions between these two cognitive processes and inhibition, are capable of predicting safety behavior over longer periods.

The second limitation concerns the dependent variables, which are self-reported behaviors. Considering the social desirability biases of self-reported measures, people tend to over-report their safety attitude and safety behaviors. Therefore, these results may be criticized for common method bias, and the relationship between these dependent variables and their predictors may be attenuated [46]. However, we employed several procedures suggested by Tsui, Ashford, Clair, and Xin (1995) to avoid or to examine the common method variance [63]. First, we assessed safety behaviors after safety attitude based on previous studies in order to minimize common method bias. Second, we used a single factor test [64] to check if all the items involved belong to one factor. The result indicated that the first unrotated factor did not account for the majority of the covariance. Third, CFA showed that a two-factor model of self-reported predictors is superior to the one-factor model. Although the last two statistical methods cannot eliminate common method bias, both indicated that the bias may not be a serious problem. One may also argue that the outcome measure only gets at self-reported measures of safety behaviors, and may not be very strong at parts governed by automatic components. However, the term “automatic” refers to an individual's lack of awareness of the activation and expression of their association, which does not mean they will not recognize the behavior. In other words, an individual knows explicitly what they do rather than why they do it. Further research can use multi-resource data, such as objective records and behavioral observation to examine the relationships suggested by the current research.

Finally, several methodological limitations of the IAT measures should be noted. General cognitive skills and executive functions have been proposed to directly influence the estimates of associations provided by IAT [65], [66]. However, we use the D measure as the index of IAT [56], which can eliminate the effect of method-specific variance [67]. Given that the IAT result in the present study is not positively correlated with the Stroop effect task, potential contamination between the predictor and the moderator in the current study is unlikely.

## Conclusion

This study examined the social cognitive mechanisms underlying safety behaviors based on a dual-process model of controlled and automatic cognitions within a field setting. Results demonstrate that both controlled cognitive and automatic cognitive processes can predict safety behavior. Moreover, study of additional moderators revealed that the capacity of inhibition contributes to safety behavior determinations. The safety behaviors of employees with lower inhibitory control are driven more by automatic safety cognition, whereas the behaviors of employees with higher inhibitory control are mainly guided by self-reported safety attitude. Safety-priority organizations should pay attention to both controlled and automatic cognitions, as well as individual differences in their inhibition level in safety management.

## Supporting Information

Table S1 Factor Loadings for Items of Self-reported Measures.
(DOC)

Table S2 IAT Stimuli.
(DOC)
