# Supplementary material for: Controlled versus Automatic Processes: Which Is Dominant to Safety? The Moderating Effect of Inhibitory Control
Source: PLoS One. 2014 Feb 10;9(2):e87881. doi: 10.1371/journal.pone.0087881 (PMC3919723; doi:10.1371/journal.pone.0087881)
Supplement: Table S1 — Factor Loadings for Items of Self-reported Measures. (DOC) [file pone.0087881.s001.doc]

**Table S1.** Factor Loadings for Items of Self-reported Measures

| Variable and item | Factor loading |
| --- | --- |
| General safety attitude |  |
| Safety specific jobs should always get done | .79 |
| Safety should have a high priority | .61 |
| Safety training should be given top priority over other training | .74 |
| Organizations should have defined safety objectives | .79 |
| Safety issues should be assigned high priority in management meetings | .75 |
| Safety compromise |  |
| Sometimes it is necessary to take risks to get a job done | .86 |
| Sometimes it is necessary to take shortcuts | .77 |
| Sometimes it is necessary to depart from safety requirements for the sake of production | .70 |
| Safety compliance |  |
| I use all the necessary safety equipment to do my job | .84 |
| I use the correct safety procedures for carrying out my job | .81 |
| I ensure the highest levels of safety when I carry out my job | .76 |
| Safety participation |  |
| I promote the safety program within the organization | .73 |
| I put in extra effort to improve the safety of the workplace | .90 |
| I voluntarily carry out tasks or activities that help to improve workplace safety | .91 |
