# Supplementary material for: Controlled versus Automatic Processes: Which Is Dominant to Safety? The Moderating Effect of Inhibitory Control
Source: PLoS One. 2014 Feb 10;9(2):e87881. doi: 10.1371/journal.pone.0087881 (PMC3919723; doi:10.1371/journal.pone.0087881)
Supplement: Table S2 — IAT Stimuli. (DOC) [file pone.0087881.s002.doc]

**Table S2.** IAT Stimuli

| Positive stimuli |  | Negative stimuli |
| --- | --- | --- |
| Gain |  | Loss |
| Win |  | Failure |
| Success |  | Mistake |
| Benefit |  | Cost |
| Achieve |  | Penalty |
| Reward |  | Lose |
| Profit |  | Waste |
